# PCSK9 promotes tumor growth by inhibiting tumor cell apoptosis in hepatocellular carcinoma

**DOI:** 10.1186/s40164-021-00218-1

**Published:** 2021-03-31

**Authors:** Shi-Zhe Zhang, Xiao-Dong Zhu, Long-Hai Feng, Xiao-Long Li, Xue-Feng Liu, Hui-Chuan Sun, Zhao-You Tang

**Affiliations:** 1 Department of Liver Surgery and Transplantation, Liver Cancer Institute and Zhongshan Hospital, Fudan University, Shanghai, 200032 China; 2 Key Laboratory of Carcinogenesis and Cancer Invasion of Ministry of Education, Shanghai, 200032 China

**Keywords:** Hepatocellular carcinoma, PCSK9, Apoptosis, FASN

## Abstract

**Background:**

Proprotein convertase subtilisin/kexin type 9 (PCSK9), one of the key enzymes in the process of lipid transport, is involved in the disease progression of various types of tumors. This article is to study the role of PCSK9 in the progression of hepatocellular carcinoma (HCC).

**Methods:**

Immunohistochemistry was used to assess the expression of PCSK9 in tumor specimens from 105 HCC patients who underwent curative resection. Western blotting and quantitative real-time PCR were used to test the protein and mRNA expression levels in HCC cell lines. Cell Counting Kit-8 (CCK-8) and clone formation assays were performed to evaluate the proliferation ability of different kinds of cells in vitro. Flow cytometry was used to analyze cell cycle distribution and apoptosis rate. A xenograft model was established to study the effect of PCSK9 on HCC growth in vivo. TUNEL and immunofluorescence assays were used to detect cell apoptosis.

**Results:**

High expression of PCSK9 in tumor tissues was related to microvascular invasion (*p* = 0.036) and large tumor size (*p* = 0.001) in HCC patients. Overall survival and disease-free survival after surgery were poor in patients with high expression of PCSK9 (*p* = 0.035 and *p* = 0.007, respectively). In vivo and in vitro experiments showed that PCSK9 promoted the growth of HCC by inhibiting cell apoptosis. A mechanistic study revealed that PCSK9 increases FASN expression, thereby inhibiting apoptosis of HCC cells via the Bax/Bcl-2/Caspase9/Caspase3 pathway.

**Conclusions:**

PCSK9 expression level in HCC is an indicator of poor prognosis for patients with HCC. FASN-mediated anti-apoptosis plays an important role in PCSK9-induced HCC progression.

## Background

Liver cancer, mostly hepatocellular carcinoma (HCC), has the fifth-highest morbidity and the second-highest mortality rate among men worldwide [1]. Although we have witnessed advances in HCC treatment in recent years, the rapid growth and high recurrence rate after curative therapy are still important factors affecting prognosis. To date, only anti-angiogenic therapy and immune-checkpoint inhibitors have shown clinically significant effects in practice.

Proprotein convertase subtilisin/kexin type 9 (PCSK9) is mainly synthesized by hepatocytes and is one of the key enzymes in lipid transport. PCSK9 causes low-density lipoprotein (LDL) accumulation in the blood by reducing the amount of low-density lipoprotein receptor (LDLR) on the cell membrane, leading to hyperlipidemia [2–4]. In addition, PCSK9 is closely related to nervous system development [5, 6], hepatocyte regeneration [7], and islet cell function regulation [8]. Recently, PCSK9 was found to inhibit cell apoptosis in a variety of tumors, such as neuroglioma [9], lung adenocarcinoma [10], melanoma [11], and neuroendocrine neoplasms [12]. However, it is still unknown whether the expression level of PCSK9 in HCC has clinical significance. In this study, we investigated the relationship between the expression level of PCSK9 in tumor cells and patient prognosis after curative surgery, and then explored the mechanisms by which PCSK9 promotes tumor growth in HCC.

## Methods

### Patients and tissue specimens

This study was approved by the Clinical Research Ethics Committee of Zhongshan Hospital, Fudan University, Shanghai, China, and all patients signed informed consent forms. Tissue microarrays (TMAs) were composed of specimens from 105 HCC patients who underwent curative liver resection from December 2009 to December 2010 at Zhongshan Hospital of Fudan University. These patients had no extrahepatic metastasis of HCC and had not received any anti-tumor treatment before surgery. Detailed methods of TMA establishment and patient follow-up were described in our previous study [13]. Overall survival (OS) was defined as the time between surgery and patient death or the last follow-up date. Disease-free survival (DFS) was defined as the time between surgery and tumor recurrence or patient death.

### Immunohistochemistry

Paraffin sections were incubated with rabbit anti-PCSK9 antibody (1:100, ProteinTech Group, Chicago, IL). Ultra-Vision Quanto Detection System HRP DAB (Thermo Fisher Scientific, CA, USA) was used to detect PCSK9 expression. The integrated optical density (IOD) of positive expression was calculated using Image-Pro Plus 6.0 software. The detailed steps were described in our previous study [14].

### Cell culture

The L02 cell line is a human hepatocyte cell line. MHCC-97H, MHCC-97L, Huh7, HepG2, PLC, HCCLM3, and SMMC7721 cell lines are different human HCC cell lines [15, 16]. All these cell lines were obtained from the Liver Cancer Institute of Fudan University (Shanghai, China). All cells were cultured in Dulbecco’s modified Eagle’s medium (DMEM; Invitrogen, Carlsbad, CA) supplemented with 10% fetal bovine serum (FBS; Gibco, Grand Island, NY) and 1% streptomycin/penicillin.

### Lentivirus transfection

Lentiviruses containing PCSK9-overexpressed or PCSK9-downregulated nucleic acid sequences and their corresponding control lentiviruses (Lv-PCSK9, Lv-Vector, Lv-shPCSK9, Lv-shVector) were constructed by Genomeditech (Shanghai, China). Before transfection, approximately 3 × 10^5^ tumor cells were seeded in each well of 6-well plates. Lentiviruses were added to the respective HCC cells with 1.5 ml of DMEM containing no FBS and 5 μg/ml Polybrene (Sigma, USA). Twenty-four hours later, the medium was replaced with DMEM containing 10% FBS. The efficiency of transfection was detected by qPCR and western blotting.

### Western blotting and quantitative real-time PCR assays

We extracted 30 μg of total protein from cultured cells, separated the proteins using 10% SDS-PAGE and transferred proteins onto polyvinylidene difluoride membranes. Then, we blocked proteins with 5% skim milk for 30 min and incubated them with diluted primary antibody. Primary antibodies for GAPDH, PCSK9, FASN, Bax, Bcl-2, Caspase9, and Caspase3 were purchased from Abcam (Cambridge, UK). Specific steps were performed as previously described [17]. The primers used for qRT-PCR are as follows: PCSK9, 5′-GCTGAGCTGCTCCAGTTTCT-3′ (forward) and 5′-AATGGCGTAGACACCCTCAC-3′ (reverse); GAPDH, 5′-AAGGTGAAGGTCGGAGTCAAC-3′ (forward) and 5′-GGGGTCATTGATGGCAACAATA-3′ (reverse).

### Clone formation assay

Approximately 500 individual HCCLM3 cells or 800 individual HepG2 cells were isolated and then seeded in one well of a 6-well plate. Approximately two weeks later, when colonies visible to the naked eye appeared, we fixed the cells with formalin for 30 min, stained them with 0.1% crystal violet for 15 min and then counted the colonies under a microscope (Olympus, Tokyo, Japan).

### Apoptosis assay

Flow cytometry was used to detect the apoptosis rate of HCC cells in vitro. For the TUNEL assays, a TUNEL reaction mixture (Roche, Pleasanton, CA, USA) and DAPI (ProteinTech Group) were used. Apoptotic cells appeared as red-stained cells under a fluorescence microscope (Olympus, Tokyo, Japan).

### Tumor xenograft model of nude mice

As described in the previous literature, an orthotopic tumor xenograft model was set up with 5-week-old male BALB/c nude mice obtained from the Beijing Vital River Laboratory Animal Technologies Co. Ltd and kept under specific pathogen-free conditions [18]. The mice were randomly grouped, and each group contained six mice. About 6 × 10^6^ HCCLM3 or HepG2 cells dissolved in 200 μl PBS were subcutaneously seeded in 5-week-old male BALB/c nude mice. Four weeks later, subcutaneous tumor nodules were removed from the nude mice as xenograft sources. We cut the xenograft sources into small nodules (2.0 × 2.0 × 2.0 mm^3^) and inoculated these dissected subcutaneous tumor nodules into the liver capsules of male BALB/c nude mice to establish an orthotopic transplantation model. Four weeks later, we executed the mice and removed the xenografts for further study. The calculation of tumor volume adopts the classic calculation formula V = (length × width^2^)/2.

### Statistical analysis

Continuous variables were expressed as the mean ± standard deviation or median (range) and were compared using Student’s t-test or the Mann–Whitney U-test. Categorical variables were compared using the χ2 test or Fisher’s exact test. Univariate survival analysis was performed using the Kaplan–Meier method. The significance of each variable for predicting OS and DFS was analyzed using Cox proportional hazards regression models. All statistical tests were two-tailed, and *p* < 0.05 was defined as a significant difference. SPSS v19.0 (IBM Inc, USA) was used as the statistical analysis software.

## Results

### High expression of PCSK9 in HCC cells correlated with poor prognosis of HCC patients

**Fig. 1:**
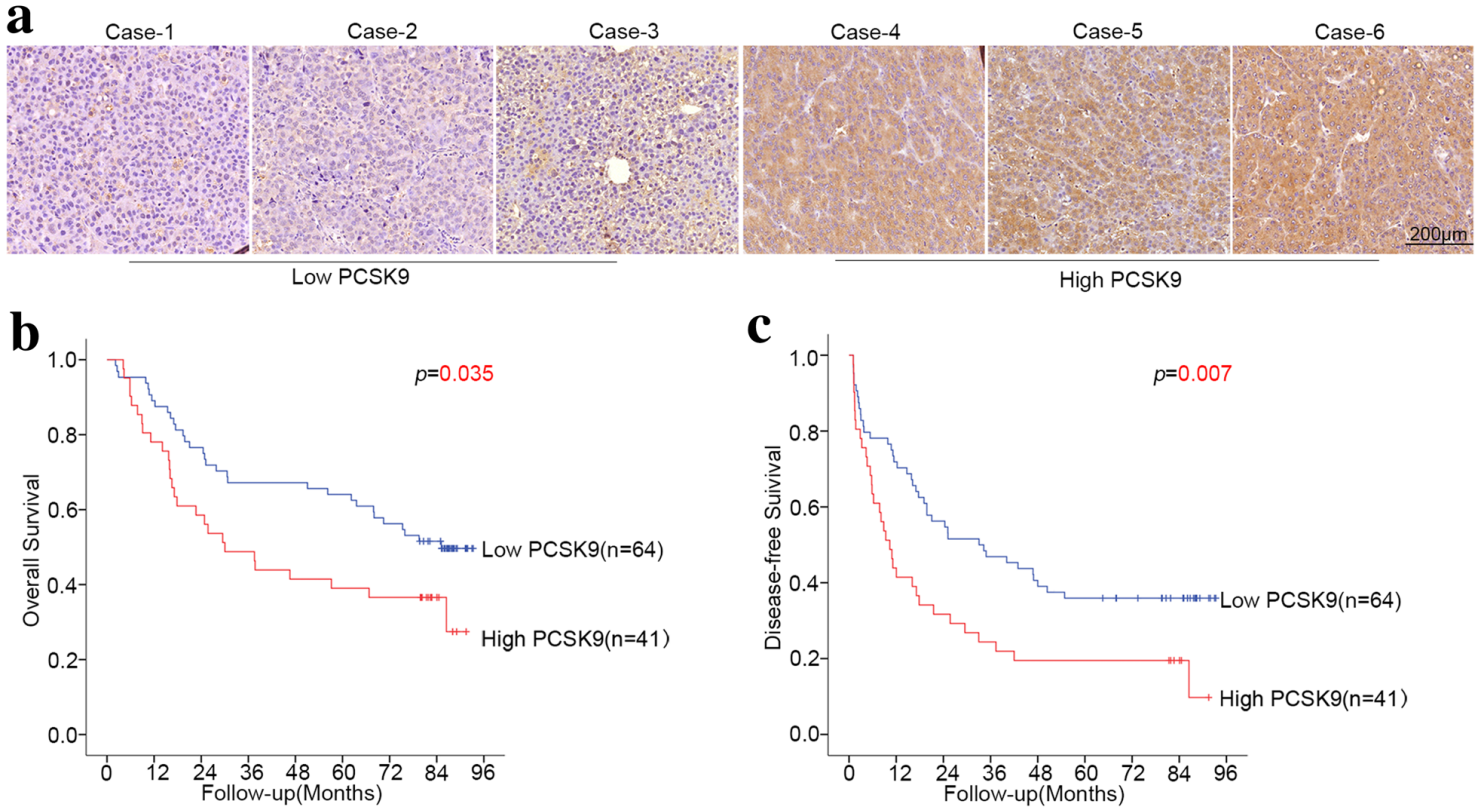
Intratumoral PCSK9 expression was associated with prognosis in HCC patients who underwent curative resection. **a** Immunohistochemistry staining of liver cancer tissues from different HCC patients. Typical cases with low or high PCSK9 expression are shown. Kaplan–Meier analysis showed that patients with high PCSK9 expression had poor overall survival (*p* = 0.035) (**b**) and poor disease-free survival (*p* = 0.007) (**c**)

We selected 105 consecutive patients who underwent radical resection of HCC at Zhongshan Hospital from December 2009 to December 2010 and constructed a tissue microarray (TMA) with their tumor tissues. The expression level of PCSK9 in different HCC patient tissues varies greatly (Fig. 1a). The distribution of IOD values among this cohort of patients is normally distributed (lnIOD = 16.74 ± 1.59). Cutoff Finder [19] was used to find an ideal cutoff value to stratify patients with different prognoses. Then, we divided the patients into a high-PCSK9 group (n = 41) and low-PCSK9 group (n = 64). The expression of PCSK9 in tumor tissues of HCC patients was associated with large tumor size (*p* = 0.001) and microvascular invasion (*p* = 0.036). The expression of PCSK9 was not associated with patient age, gender, serum AFP level, number of tumors, or tumor cell differentiation (*p* > 0.05 for all) (Table 1).

**Table 1:** Relationship between intratumoral PCSK9 expression and clinicopathological features of HCC patients

Variable	PCSK9 expression		*p*
	Low	High	
Age (years)			
≤ 50	23	14	0.851
> 50	41	27	
Gender			
Female	10	5	0.624
Male	54	36	
AFP (ng/ml)			
≤ 20	25	19	0.461
> 20	39	22	
Number of tumors			
Single	44	28	0.961
Multiple	20	13	
Tumor size (cm)			
≤ 5	43	14	0.001
> 5	21	27	
Tumor cell differentiation			
I–II	45	30	0.752
III–IV	19	11	
Microvascular invasion			
No	32	12	0.036
Yes	32	29	

Univariate analysis showed that OS was associated with serum AFP levels (*p* = 0.001), tumor size (*p* < 0.001), tumor cell differentiation (*p* = 0.008), microvascular invasion (*p* = 0.018) and PCSK9 expression (*p* = 0.035) (Table 2, Fig. 1b); DFS was associated with serum AFP levels (*p* = 0.005), number of tumors (*p* = 0.037), tumor size (*p* = 0.002), and PCSK9 expression (*p* = 0.007) (Table 2, Fig. 1c). The median OS in the low PCSK9 group was longer than that in the high PCSK9 group (80.15 vs. 30.03 months), and the median DFS in the low PCSK9 group was longer than that in the high PCSK9 group (33.67 vs. 10.37 months). Cox multivariate regression analysis indicated that serum AFP levels (*p* < 0.001), tumor size (*p* = 0.001), and PCSK9 levels (*p* = 0.049) were independently associated with OS, and serum AFP levels (*p* = 0.001), tumor number (*p* = 0.041), tumor size (*p* = 0.017), and PCSK9 expression level (*p* = 0.007) were independently associated with DFS (Table 2). Overall, high PCSK9 expression in HCC tissues is an independent risk factor for both OS and DFS in patients with HCC who underwent curative resection.

**Table 2:** Univariate and multivariate analyses of factors associated with survival and recurrence

Factor	Overall survival				Disease-free survival			
	Univariate	Multivariate			Univariate	Multivariate		
	*p*	Hazard ratio	95% CI	*p*	*p*	Hazard Ratio	95% CI	*p*
Age (years) ≤ 50 vs > 50	0.664			NA	0.381			NA
Gender Female vs Male	0.982			NA	0.972			NA
AFP (ng/ml) > 20 vs ≤ 20	0.001	2.94	1.644 −5.258	0.000	0.005	2.292	1.410 −3.724	0.001
Number of tumors Multiple vs single	0.079			NA	0.037	1.668	1.021 −2.723	0.041
Tumor size (cm) > 5 vs ≤ 5	0.000	2.572	1.460 −4.529	0.001	0.002	1.812	1.115 −2.946	0.017
Tumor cell differentiation III–IV vs I–II	0.008	1.699	0.975 –2.961	0.061	0.248			NA
Microvascular invasion Yes vs No	0.018	1.246	0.690 –2.251	0.465	0.057			NA
PCSK9	0.035	1.788	1.003 –3.185	0.049	0.007	1.956	1.199 −3.192	0.007
High vs Low								

NA: not applicable

### PCSK9 promoted cell proliferation in vitro

**Fig. 2:**
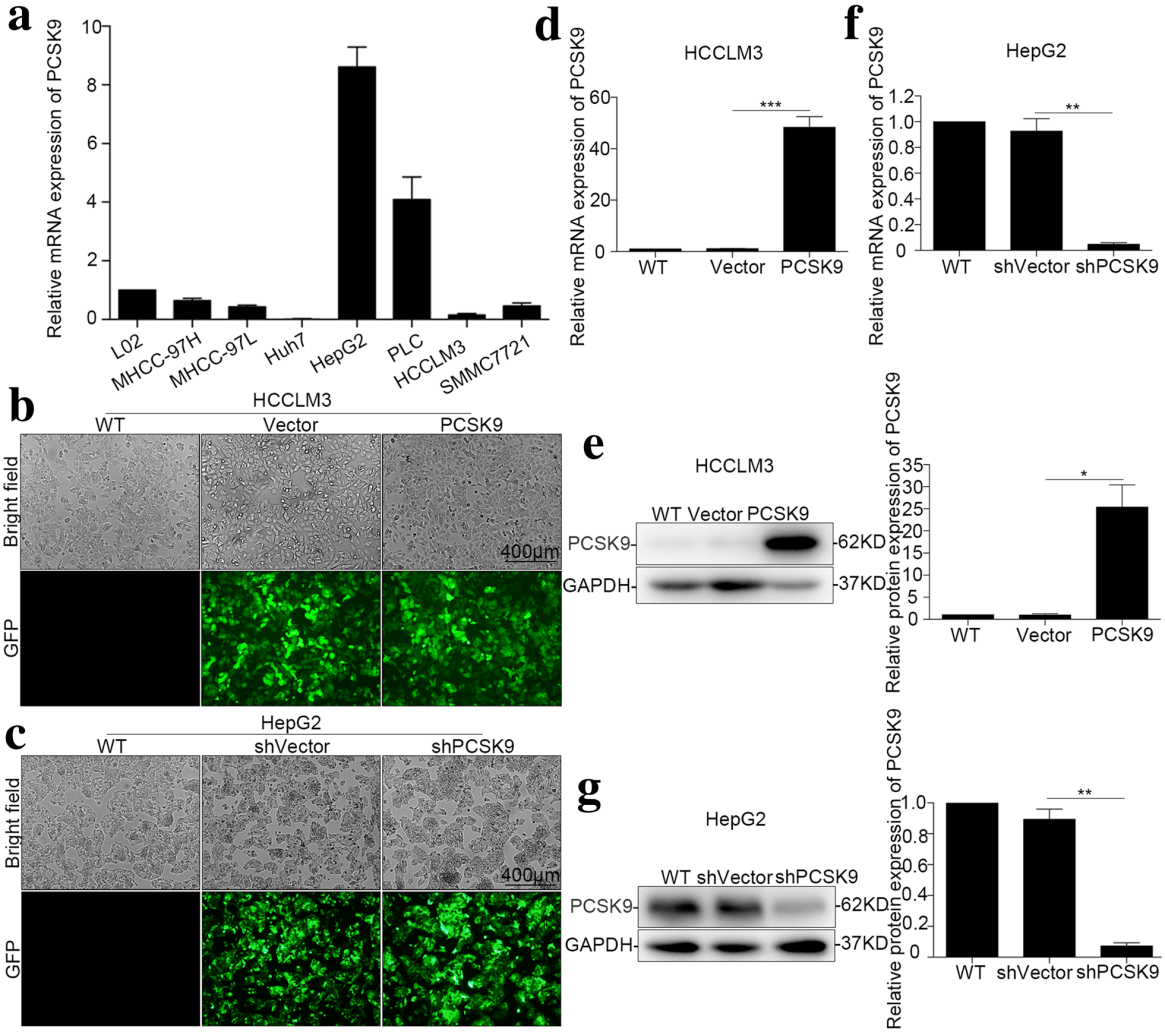
Construction of HCC cell lines with overexpression or downregulation of PCSK9. **a** Relative mRNA expression level of PCSK9 in HCC cell lines and a normal hepatocyte cell line (L02). **b** Transfection efficiency of PCSK9-overexpressing virus in the HCCLM3 cell line. **c** Transfection efficiency of PCSK9-knockdown virus in the HepG2 cell line. **d** Relative mRNA expression level of PCSK9 in the HCCLM3 cell line (*p* < 0.001). **e** Western blot images and summarized data showing that PCSK9 was successfully overexpressed in the HCCLM3 cell line (*p* = 0.014). **f** Relative mRNA expression level of PCSK9 in the HepG2 cell line (*p* = 0.004). **g** Western blot images and summarized data showing that PCSK9 was successfully downregulated in the HepG2 cell line (*p* = 0.001). *p < 0.05, **p < 0.01, ***p < 0.001

Because the expression of PCSK9 was low in HCCLM3 cells and high in HepG2 cells (Fig. 2a), we chose these two cell lines for further study. Stable overexpression of PCSK9 in HCCLM3 cells, downregulation of PCSK9 in HepG2 cells, and the corresponding vector control were established. Lv-Vector, Lv-PCSK9, Lv-shVector and Lv-shPCSK9 with green fluorescent protein (GFP) were used for transfection of lentivirus. After transfection, strong GFP expression was observed in HCC cells under an inverted fluorescence microscope, and the efficiency of lentiviral transfection was over 90% (Fig. 2b, c). Then, qRT-PCR and Western blotting were used to verify the efficiency of PCSK9 overexpression or downregulation (Fig. 2d–g).

**Fig. 3:**
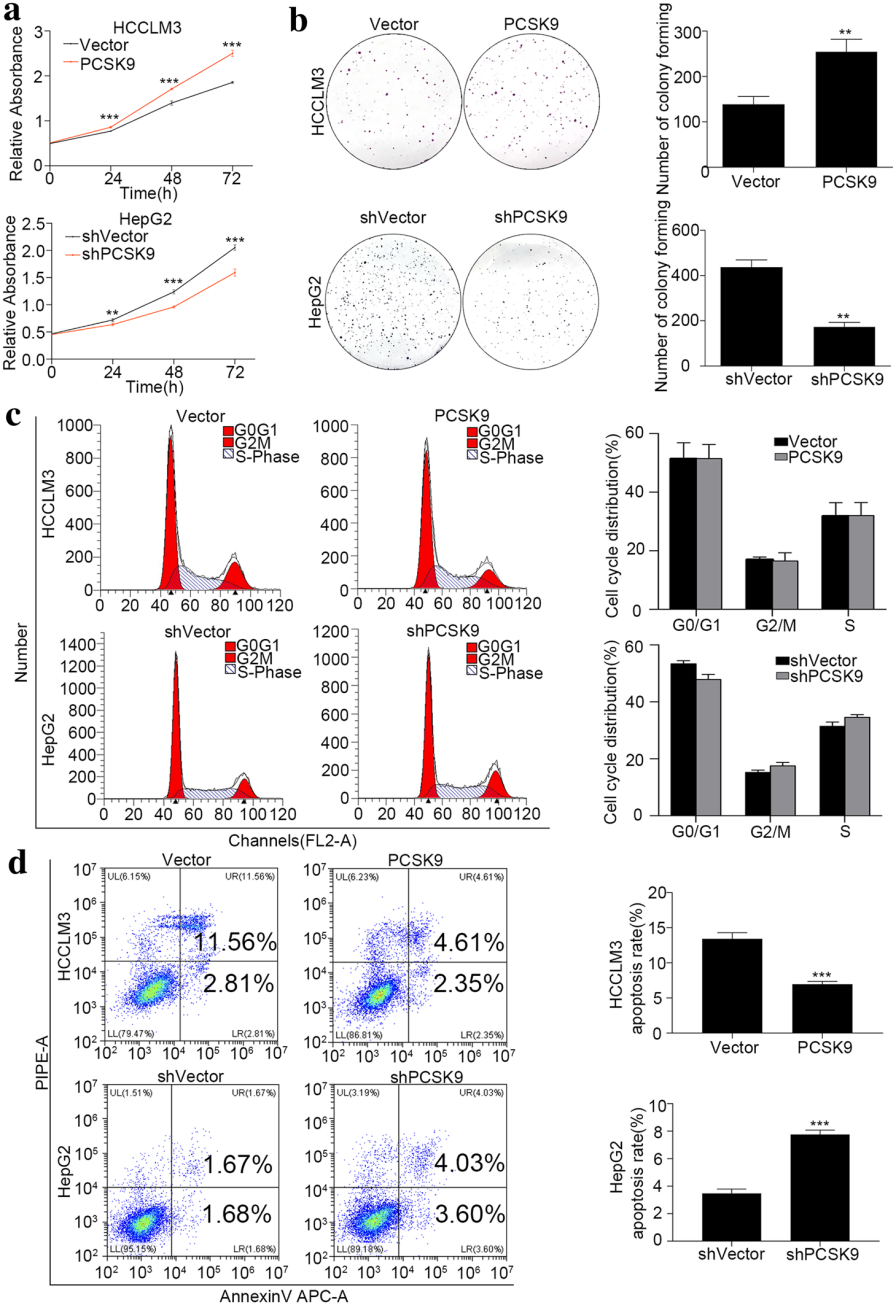
PCSK9 inhibited apoptosis and promoted the proliferation of HCC cells in vitro. **a** CCK8 assay for cell proliferation of HCCLM3-PCSK9 cells and HepG2-shPCSK9 cells compared with their vector control. PCSK9 promoted HCC cell proliferation. **b** Clone formation and summarized data for HCCLM3-PCSK9 cells (*p* = 0.005) and HepG2-shPCSK9 cells (*p* = 0.001) compared with their vector controls. PCSK9 promoted clone formation. **c** Flow cytometry detection of the cell cycle distribution of HCCLM3-PCSK9 cells and HepG2-shPCSK9 cells compared with their vector controls. PCSK9 had no significant influence on the G2/M phase in cell cycle distribution (*p* > 0.05). **d** Flow cytometry apoptosis detection to determine the cell apoptosis rates of HCCLM3-PCSK9 cells (*p* < 0.001) and HepG2-shPCSK9 cells (*p* < 0.001) compared with their vector controls. PCSK9 decreased the apoptosis rate of HCC cells in vitro. **p < 0.01, ***p < 0.001

To clarify the role of PCSK9 in HCC proliferation, CCK8 proliferation assay was used. In the HCCLM3 cell line, compared with the control (HCCLM3-Vector), the proliferation of PCSK9-overexpressing cells (HCCLM3-PCSK9) was significantly enhanced (*p* < 0.001), and in the HepG2 cell line, the proliferation of PCSK9-downregulated (HepG2-shPCSK9) cells was significantly decreased compared with that of the control cells (HepG2-shVector) (*p* < 0.01) (Fig. 3a). The plate cloning experiment revealed that the clone formation ability of HCCLM3-PCSK9 cells was stronger than that of HCCLM3-Vector cells (*p* = 0.005), and the clone formation ability of HepG2-shPCSK9 cells was weaker than that of HepG2-shVector cells (*p* = 0.001) (Fig. 3b).

To further explore the mechanisms underlying the effects of PCSK9 on HCC cell proliferation, we analyzed the cell cycle distribution and apoptosis in cultured cells. In the cell cycle analysis, the G2/M phase ratio of HCCLM3-PCSK9 cells was similar to that of HCCLM3-Vector cells (16.51 ± 2.81% vs. 16.93 ± 0.93%, *p* = 0.82). Additionally, the G2/M phase ratio of HepG2-shPCSK9 cells was similar to that of HepG2-shVector cells (17.52 ± 1.18% vs. 15.23 ± 0.80%, *p* = 0.06) (Fig. 3c). In the cell apoptosis analysis, the proportion of apoptotic HCCLM3-PCSK9 cells was significantly lower than that of HCCLM3-Vector cells (6.96 ± 0.39% vs*.* 13.43 ± 0.86%, *p* < 0.001), and the proportion of apoptotic HepG2-shPCSK9 cells was significantly higher than that of HepG2-shVector cells (7.77 ± 0.31% vs. 3.47 ± 0.31% *p* < 0.001) (Fig. 3d). These data indicated that PCSK9 promoted the proliferation of tumor cells by preventing apoptosis rather than by directly promoting cell division.

### PCSK9 promoted HCC progression in vivo

**Fig. 4:**
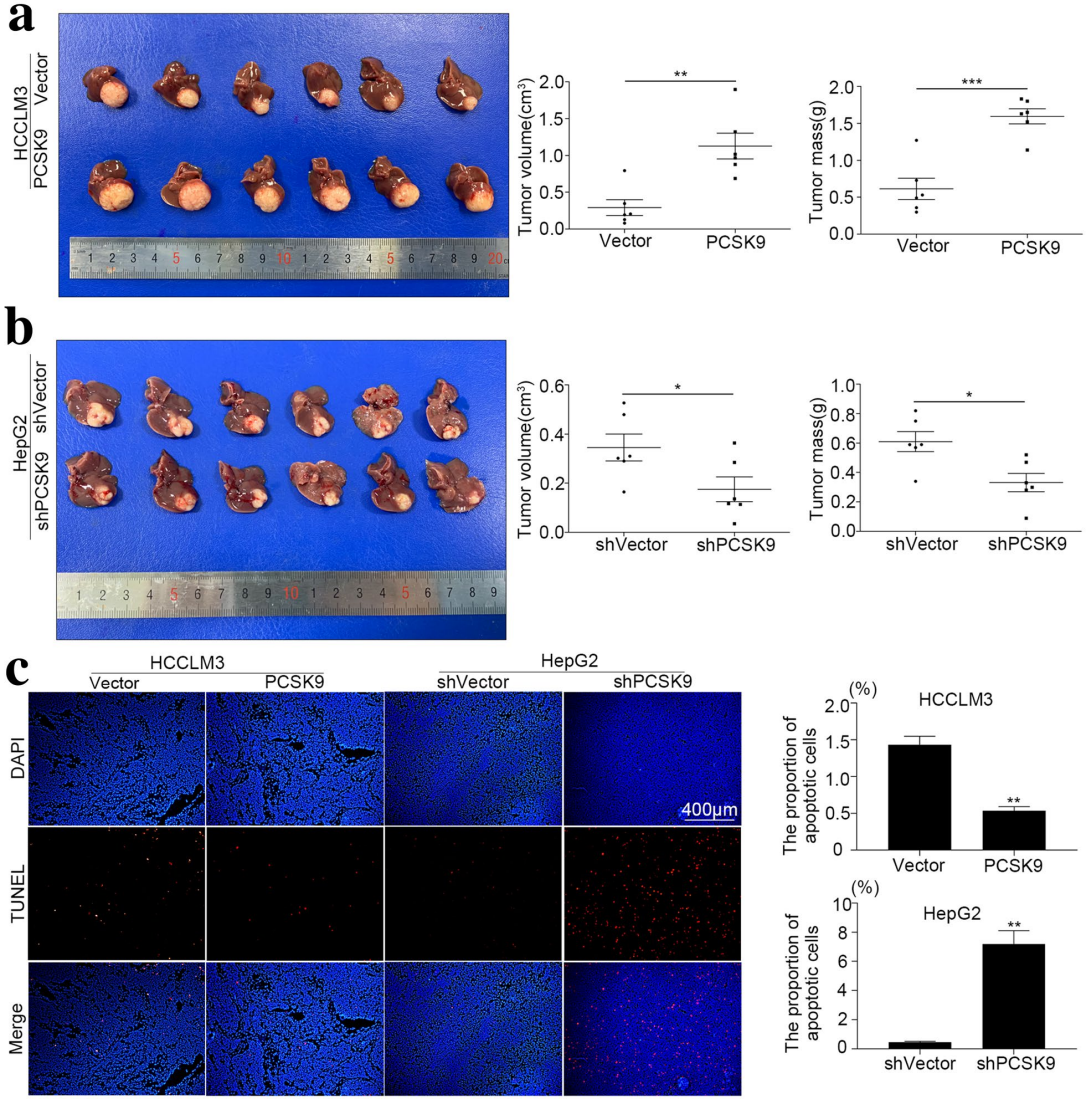
PCSK9 promoted HCC growth in vivo. We constructed orthotopic human HCC xenograft mouse models with HCC cell lines with overexpression or downregulation of PCSK9. **a** Comparison of tumor volume (0.29 ± 0.26 vs. 1.13 ± 0.43 cm^3^, n = 6, *p* = 0.001) and tumor mass (0.61 ± 0.35 vs. 1.59 ± 0.25 g, n = 6, *p* < 0.001) of the HCCLM3-Vector and HCCLM3-PCSK9 groups. **b** Comparison of tumor volume (0.34 ± 0.13 vs. 0.18 ± 0.12 cm^3^, n = 6, *p* = 0.045) and tumor mass (0.61 ± 0.17 vs. 0.33 ± 0.15 g, n = 6, *p* = 0.013) of the HepG2-shVector and HepG2-shPCSK9 groups. **c** TUNEL fluorescence of liver cancer from BALB/c nude mice orthotopically implanted with HCCLM3 or HepG2 cells and their control groups. The apoptosis rate was lower in the HCCLM3-PCSK9 group than in the control group (*p* = 0.001). The apoptosis rate was higher in the HepG2-shPCSK9 group than in the control group (*p* = 0.006). *p < 0.05, **p < 0.01, ***p < 0.001

To study the effects of PCSK9 on tumor growth in vivo, we constructed orthotopic human HCC xenograft mouse models using HCC cell lines with PCSK9 overexpression or downregulation. In the HCCLM3-PCSK9 group, the volume and weight of xenografts were significantly greater than those in the control group (volume: 1.13 ± 0.43 vs. 0.29 ± 0.26 cm^3^, *p* = 0.001, weight: 1.59 ± 0.25*.* 0.61 ± 0.35 g, *p* < 0.001) (Fig. 4a). In the HepG2-shPCSK9 group, tumor volume and tumor weight were significantly lower than those in the control group (volume: 0.18 ± 0.12 vs. 0.34 ± 0.13 cm^3^, *p* = 0.045, weight: 0.33 ± 0.15 vs. 0.61 ± 0.17 g, *p* = 0.013) (Fig. 4b). TUNEL staining of formalin-fixed paraffin-embedded xenograft tumors confirmed that the apoptosis of tumor cells was inhibited after overexpression of PCSK9 and increased after downregulation of PCSK9 (Fig. 4c). The results of in vivo experiments confirmed that PCSK9 promoted the growth of HCC and was associated with tumor cell apoptosis.

### PCSK9 inhibits apoptosis of HCC cells via the FASN/Bax/Bcl-2/Caspase9/Caspase3 pathway

**Fig. 5:**
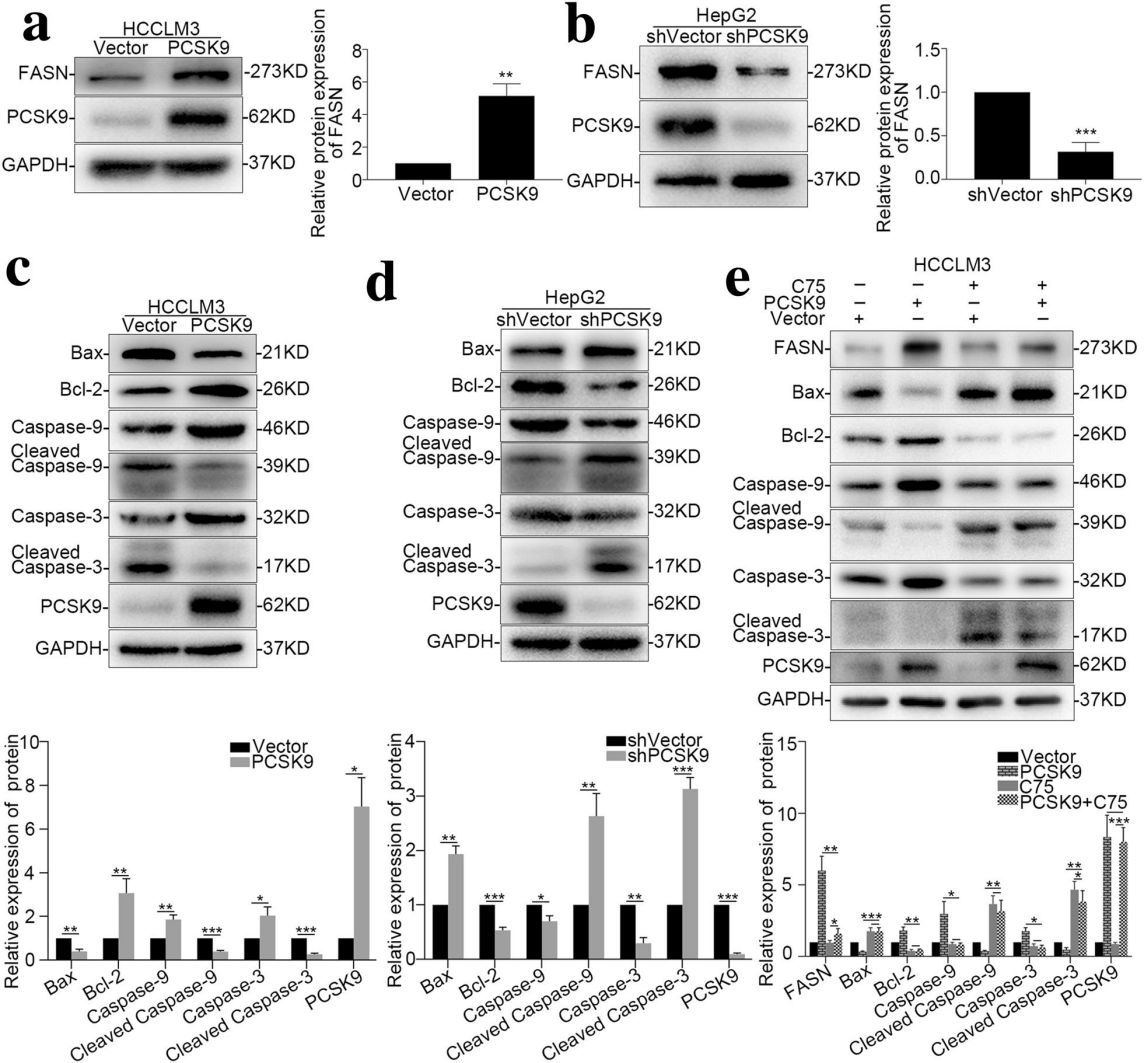
The FASN/Bax/Bcl-2/Caspase9/Caspase3 pathway is involved in the regulation of PCSK9 on HCC cell apoptosis. **a** Western blot images and summarized data showing that FASN is overexpressed in the HCCLM3-PCSK9 cell line compared with its control group (*p* = 0.001). **b** Western blot images and summarized data showing that FASN is downregulated in the HepG2-shPCSK9 cell line compared with its control group (*p* < 0.001). **c** The Bax/Bcl-2/Caspase9/Caspase3 apoptosis signaling pathway-associated proteins were detected by Western blot in HCCLM3-PCSK9 cells and controls. **d** The Bax/Bcl-2/Caspase9/Caspase3 apoptotic signaling pathway-associated proteins were detected by Western blot in HepG2-shPCSK9 cells and controls. **e** Specifically blocking FASN with C75 altered the expression of Bax/Bcl-2/Caspase9/Caspase3 apoptotic signaling pathway-associated proteins in the HCCLM3-PCSK9 cell line. *p < 0.05, **p < 0.01, ***p < 0.001

Fatty acid synthase (FASN) is one of the key enzymes in the de novo synthesis of fatty acids, and it also plays an important role in cell apoptosis in various kinds of tumors [20–23]. A FASN-specific inhibitor can effectively promote apoptosis of tumor cells [24]. Previous reports confirmed that FASN has a “coexpression” relationship with PCSK9 in HCC [25]. We verified the expression level of FASN in our PCSK9-overexpressing and PCSK9-downregulated cell lines with western blotting and found that overexpression of PCSK9 promoted the expression of FASN in HCC cell lines (*p* = 0.001) (Fig. 5a) and that downregulation of PCSK9 reduced the expression levels of FASN (*p* = 0.008) (Fig. 5b).

To further determine the mechanism by which PCSK9 inhibits HCC cell apoptosis, we examined the changes of key molecules in the apoptosis pathway. Bcl-2, which inhibits apoptosis, was upregulated in HCCLM3 cells with PCSK9 overexpression in comparison with the control cells, whereas Bax, cleaved Caspase 9, and cleaved Caspase 3, which promote apoptosis, were downregulated (*p* < 0.05 for all) (Fig. 5c). In HepG2 cells with PCSK9 downregulation, Bcl-2 was downregulated, and Bax, cleaved Caspase-9 and cleaved Caspase-3 were upregulated (*p* < 0.05 for all) (Fig. 5d).

Finally, the HCCLM3-PCSK9 cell line was treated with a specific inhibitor of FASN (C75), and C75 reversed the inhibitory effect of PCSK9. In HCCLM3-PCSK9 cells, after treatment with C75, the expression of Bcl-2 was downregulated, and the expression of Bax, cleaved Caspase-9, and cleaved Caspase-3 was upregulated (*p* < 0.05 for all), but between HCCLM3-Vector and HCCLM3-PCSK9 cells treated with C75, no significant difference was detected in the apoptotic pathway (Fig. 5e). These data suggested that PCSK9 inhibited apoptosis of HCC cells through the FASN/Bax/Bcl-2/Caspase9/Caspase3 pathway.

## Discussion

In this study, we found that high expression of PCSK9 in HCC is related to microvascular invasion and large tumor size and is an independent risk factor for both OS and DFS in patients with HCC who underwent curative resection. In vivo and in vitro experiments showed that PCSK9 promoted the growth of HCC by inhibiting cell apoptosis via the FASN/Bax/Bcl-2/Caspase9/Caspase3 pathway.

HCC is a malignant tumor with rapid proliferation, and the majority of HCC patients are diagnosed with advanced stage disease. Some researchers have found that HCC patients with hyperlipidemia have faster HCC progression and poorer prognosis [26–28]. As a critical regulatory molecule in the lipid transport process, PCSK9 interferes with the utilization of LDL and triglycerides by degrading LDLR [2, 3]. In addition to regulating lipid transport, PCSK9 has many other effects on cell functions, such as in the processes of viral infection [29–31], insulin resistance [8, 32], development of the central nervous system [5], tumor apoptosis [9, 10] and tumor immunity [33, 34]. Therefore, we hypothesized that PCSK9 might play a role in the progression of HCC. We found that a high level of PCSK9 expression is associated with poor prognosis in HCC, which coincides with the relationship between hyperlipidemia and HCC.

The tumor growth rate is a function of both cell proliferation and apoptosis. To investigate the relationships between PCSK9 and cell proliferation and apoptosis, we constructed HCC cell lines with overexpression or downregulation of PCSK9. Upon analyzing the results of the CCK8 proliferation assay and plate cloning experiment, we found that PCSK9 promoted the proliferation of HCC cells. However, in the cell cycle analysis, G2/M phase arrest was not found after overexpressing or downregulating PCSK9, which means that PCSK9 has no significant effect on cell division in HCC cells. Therefore, we next explored the effect of PCSK9 on apoptosis and found an inhibitory effect. Not only in HCC, researchers have confirmed that PCSK9 can influence the apoptosis of other tumors, such as neuroglioma [9], lung adenocarcinoma [10], melanoma [11], and neuroendocrine neoplasms [12]. To verify the promotion of PCSK9 on tumor growth in vivo, we established liver orthotopic human liver cancer xenograft model in BALB/c nude mice and obtained a consistent conclusion.

FASN is a key enzyme in the de novo synthesis process of fatty acids. Additionally, it plays an important role in the apoptosis of many kinds of tumors. For example, FASN can promote the growth of breast cancer [21] and prostate cancer cells [22]. It can also inhibit the apoptosis of HCC cell lines [23], and its inhibitors can promote the apoptosis of breast cancer cells and slow the growth of breast cancer [24]. Lee and colleagues found that FASN and PCSK9 have a “coexpression” relationship in HCC [25]. Maria found that PCSK9 mRNA is positively correlated with FASN mRNA [35]. We evaluated the expression of PCSK9 and FASN in our experimental system and found that PCSK9 promotes the expression of FASN. Blocking of FASN can reduce the anti-apoptotic effect but does not affect PCSK9 expression. These findings suggest that FASN is downstream of PCSK9 in the apoptosis regulatory pathway.

The Bax/Bcl-2/Caspase9/Caspase3 pathway is a classic apoptosis pathway. Bcl-2 is considered to be a key molecule that inhibits apoptosis. Bax, cleaved Caspase9, and cleaved Caspase3 are deemed to be key molecules that promote apoptosis. After overexpression or downregulation of PCSK9, the expression of certain molecules involved in the Bax/Bcl-2/Caspase9/Caspase3 apoptosis pathway changed significantly, which is consistent with the conclusion that PCSK9 inhibits HCC cell apoptosis in vitro and in vivo. After treatment with C75, Bcl-2 was downregulated, and Bax, cleaved Caspase9 and cleaved Caspase3 were upregulated in HCCLM3-PCSK9 cell lines, but no significant difference was detected in this apoptotic pathway between HCCLM3-Vector and HCCLM3-PCSK9 cells treated with C75, indicating that the FASN/Bax/Bcl-2/Caspase9/Caspase3 apoptosis pathway plays an important role in the regulation of HCC apoptosis by PCSK9.

However, our research has some aspects worth further exploring. In the process of hyperlipidemia, PCSK9 acts mainly through extracellular pathways, but in these experiments, we studied the role of PCSK9 in inhibiting the apoptosis of HCC cells through intracellular pathways. We will explore whether PCSK9 affects HCC through extracellular pathways in the next stage of our research. To date, two kinds of monoclonal antibodies against PCSK9 are in clinical use as lipid-lowering drugs: evolocumab and alirocumab. They have remarkable lipid-lowering effects [36, 37], long intervals of administration [38, 39], few adverse reactions [40, 41] and generally tolerable [42]. We suspect that these drugs may be useful adjuvants in the treatment of HCC, and this will be further explored in our future research.

## Conclusions

This study demonstrated that PCSK9 promoted the growth of HCC, and FASN-mediated anti-apoptosis played an important role in this process. The expression of PCSK9 in tumors correlated with poor prognosis in HCC patients after curative resection and was an independent risk factor for OS and DFS, which indicates the potential of PCSK9 as a prognostic marker for HCC.

## Data Availability

The data used during the current study are available from the corresponding author on reasonable request.

## References

[1] Bray F, Ferlay J, Soerjomataram I (2018). Global cancer statistics 2018: GLOBOCAN estimates of incidence and mortality worldwide for 36 cancers in 185 countries. CA Cancer J Clin.

[2] Huang S, Henry L, Ho YK (2010). Mechanism of LDL binding and release probed by structure-based mutagenesis of the LDL receptor. J Lipid Res.

[3] Park SW, Moon YA, Horton JD (2004). Post-transcriptional regulation of low density lipoprotein receptor protein by proprotein convertase subtilisin/kexin type 9a in mouse liver. J Biol Chem.

[4] Lo Surdo P, Bottomley MJ, Calzetta A (2011). Mechanistic implications for LDL receptor degradation from the PCSK9/LDLR structure at neutral pH. EMBO Rep.

[5] Poirier S, Prat A, Marcinkiewicz E (2006). Implication of the proprotein convertase NARC-1/PCSK9 in the development of the nervous system. J Neurochem.

[6] Bingham B, Shen R, Kotnis S (2006). Proapoptotic effects of NARC 1 (= PCSK9), the gene encoding a novel serine proteinase. Cytometry A.

[7] Zaid A, Roubtsova A, Essalmani R (2008). Proprotein convertase subtilisin/kexin type 9 (PCSK9): hepatocyte-specific low-density lipoprotein receptor degradation and critical role in mouse liver regeneration. Hepatology.

[8] Awan Z, Delvin EE, Levy E (2013). Regional distribution and metabolic effect of PCSK9 insLEU and R46L gene mutations and apoE genotype. Can J Cardiol.

[9] Piao MX, Bai JW, Zhang PF (2015). PCSK9 regulates apoptosis in human neuroglioma u251 cells via mitochondrial signaling pathways. Int J Clin Exp Pathol.

[10] Xu X, Cui Y, Cao L (2017). PCSK9 regulates apoptosis in human lung adenocarcinoma A549 cells via endoplasmic reticulum stress and mitochondrial signaling pathways. Exp Ther Med.

[11] Sun X, Essalmani R, Day R (2012). Proprotein convertase subtilisin/kexin type 9 deficiency reduces melanoma metastasis in liver. Neoplasia.

[12] Bai J, Na H, Hua X (2017). A retrospective study of NENs and miR-224 promotes apoptosis of BON-1 cells by targeting PCSK9 inhibition. Oncotarget.

[13] Shi WK, Zhu XD, Wang CH (2018). PFKFB3 blockade inhibits hepatocellular carcinoma growth by impairing DNA repair through AKT. Cell Death Dis.

[14] Wang CH, Zhu XD, Ma DN (2017). Flot2 promotes tumor growth and metastasis through modulating cell cycle and inducing epithelial-mesenchymal transition of hepatocellular carcinoma. Am J Cancer Res.

[15] Tian J, Tang ZY, Ye SL (1999). New human hepatocellular carcinoma (HCC) cell line with highly metastatic potential (MHCC97) and its expressions of the factors associated with metastasis. Br J Cancer.

[16] Li Y, Tian B, Yang J (2004). Stepwise metastatic human hepatocellular carcinoma cell model system with multiple metastatic potentials established through consecutive in vivo selection and studies on metastatic characteristics. J Cancer Res Clin Oncol.

[17] Qin CD, Ma DN, Zhang SZ (2018). The Rho GTPase Rnd1 inhibits epithelial-mesenchymal transition in hepatocellular carcinoma and is a favorable anti-metastasis target. Cell Death Dis.

[18] Ma DN, Chai ZT, Zhu XD (2016). MicroRNA-26a suppresses epithelial-mesenchymal transition in human hepatocellular carcinoma by repressing enhancer of zeste homolog 2. J Hematol Oncol.

[19] Budczies J, Klauschen F, Sinn BV (2012). Cutoff Finder: a comprehensive and straightforward Web application enabling rapid biomarker cutoff optimization. PLoS ONE.

[20] Pizer ES, Wood FD, Heine HS (1996). Inhibition of fatty acid synthesis delays disease progression in a xenograft model of ovarian cancer. Cancer Res.

[21] Pizer ES, Jackisch C, Wood FD (1996). Inhibition of fatty acid synthesis induces programmed cell death in human breast cancer cells. Cancer Res.

[22] Kridel SJ, Axelrod F, Rozenkrantz N (2004). Orlistat is a novel inhibitor of fatty acid synthase with antitumor activity. Cancer Res.

[23] Li L, Pilo GM, Li X (2016). Inactivation of fatty acid synthase impairs hepatocarcinogenesis driven by AKT in mice and humans. J Hepatol.

[24] Puig T, Vazquez-Martin A, Relat J (2008). Fatty acid metabolism in breast cancer cells: differential inhibitory effects of epigallocatechin gallate (EGCG) and C75. Breast Cancer Res Treat.

[25] Lee S, Zhang C, Liu Z (2017). Network analyses identify liver-specific targets for treating liver diseases. Mol Syst Biol.

[26] Hwang SJ, Lee SD, Chang CF (1992). Hypercholesterolaemia in patients with hepatocellular carcinoma. J Gastroenterol Hepatol.

[27] Chang PE, Ong WC, Lui HF (2013). Epidemiology and prognosis of paraneoplastic syndromes in hepatocellular carcinoma. ISRN Oncol.

[28] Qu Q, Wang S, Chen S (2014). Prognostic role and significance of paraneoplastic syndromes in hepatocellular carcinoma. Am Surg.

[29] Meredith LW, Wilson GK, Fletcher NF (2012). Hepatitis C virus entry: beyond receptors. Rev Med Virol.

[30] Labonte P, Begley S, Guevin C (2009). PCSK9 impedes hepatitis C virus infection in vitro and modulates liver CD81 expression. Hepatology.

[31] Fasolato S, Pigozzo S, Pontisso P (2020). PCSK9 levels are raised in chronic HCV patients with hepatocellular carcinoma. J Clin Med.

[32] Mbikay M, Sirois F, Gyamera-Acheampong C (2015). Variable effects of gender and Western diet on lipid and glucose homeostasis in aged PCSK9-deficient C57BL/6 mice CSK9PC57BL/6. J Diabetes.

[33] Liu X, Bao X, Hu M (2020). Inhibition of PCSK9 potentiates immune checkpoint therapy for cancer. Nature.

[34] Momtazi-Borojeni AA, Nik ME, Jaafari MR (2019). Effects of immunization against PCSK9 in an experimental model of breast cancer. Arch Med Sci.

[35] Emma MR, Giannitrapani L, Cabibi D (2020). Hepatic and circulating levels of PCSK9 in morbidly obese patients: relation with severity of liver steatosis. Biochim Biophys Acta Mol Cell Biol Lipids.

[36] Giugliano RP, Desai NR, Kohli P (2012). Efficacy, safety, and tolerability of a monoclonal antibody to proprotein convertase subtilisin/kexin type 9 in combination with a statin in patients with hypercholesterolaemia (LAPLACE-TIMI 57): a randomised, placebo-controlled, dose-ranging, phase 2 study. Lancet.

[37] Stein EA, Gipe D, Bergeron J (2012). Effect of a monoclonal antibody to PCSK9, REGN727/SAR236553, to reduce low-density lipoprotein cholesterol in patients with heterozygous familial hypercholesterolaemia on stable statin dose with or without ezetimibe therapy: a phase 2 randomised controlled trial. Lancet.

[38] He XX, Zhang R, Zuo PY (2017). The efficacy advantage of evolocumab (AMG 145) dosed at 140mg every 2weeks versus 420mg every 4weeks in patients with hypercholesterolemia: evidence from a meta-analysis. Eur J Intern Med.

[39] Gaudet D, Watts GF, Robinson JG (2017). Effect of alirocumab on lipoprotein(a) over >/=1.5 years (from the Phase 3 ODYSSEY Program). Am J Cardiol.

[40] Nissen SE, Stroes E, Dent-Acosta RE (2016). Efficacy and tolerability of evolocumab vs ezetimibe in patients with muscle-related statin intolerance: the GAUSS-3 randomized clinical trial. JAMA.

[41] Robinson JG, Farnier M, Krempf M (2015). Efficacy and safety of alirocumab in reducing lipids and cardiovascular events. N Engl J Med.

[42] Dufour R, Bergeron J, Gaudet D (2017). Open-label therapy with alirocumab in patients with heterozygous familial hypercholesterolemia: results from three years of treatment. Int J Cardiol.

